# Entropic analysis of cilia-modulated slip flow of trimetallic nanofluid through electroosmotic corrugated pump in the presence of inclined magnetic field

**DOI:** 10.1038/s41598-023-30979-0

**Published:** 2023-03-06

**Authors:** Sufian Munawar, Najma Saleem, Farkhanda Afzal, Arif Mehmood, Malik Khurram Shahzad Awan, Poom Kumam

**Affiliations:** 1 Department of Applied Sciences, National Textile University, Faisalabad, 37610 Pakistan; 2 Department of Mathematics and Natural Sciences, Prince Mohammad Bin Fahd University, Khobar, 31952 Saudi Arabia; 3 MCS, National University of Science and Technology, Islamabad, Pakistan; 4 University of Science and Technology Bannu, Bannu, Pakistan; 5 Vice Deanship of Quality & Development, College of Medicine, Imam AbdulRahman Bin Faisal University, Dammam, Saudi Arabia; 6 King Mongkut’s University of Technology Thonburi (KMUTT), Bangkok, Thailand

**Keywords:** Applied mathematics, Nanoscience and technology

## Abstract

An incredible eradication of thermal indulgence is required to enhance the flow and heat transfer enhancement in micro/nanofluidic devices. In addition, the rapid transport and instantaneous mixing of colloidal suspensions of metallic particles at nanoscale are exceptionally crucial at ascendency of inertial and surface forces. To address these challenges, the present work is intended to investigate the role of trimetallic nanofluid comprising of three kinds of nano-sized granules (titanium oxide, Silica and Aluminium dioxide) with pure blood through a heated micropump in the presence of inclined magnetic field and axially implemented electric field. To ensure rapid mixing in unidirectional flow, the pump internal surface is lined-up with mimetic motile cilia with slip boundary. The embedded cilia whip in pattern due to dynein molecular motion controlled by time and produce a set of metachronal waves along the pump wall. The shooting technique is executed to compute the numerical solution. In a comparative glance it is revealed that the trimetallic nanofluid exhibits 10% higher heat transfer efficiency as compared to bi-hybrid and mono nanofluids. Moreover, the involvement of electroosmosis results in almost 17% decrease in the heat transfer rate if it values jumps from 1 to 5. The fluid temperature in case of trimetallic nanofluid is higher and thus keeps the heat transfer entropy and the total entropy lower. Furthermore, involvement of thermal radiated and momentum slip significantly contribute in reducing heat losses.

## Introduction

Microfluidic devices have tremendous uses in physiology and biochemical engineering, for instance, drug delivery micropumps, dialysis pumps, protein and DNA analysis and lab-on-chip devices etc. However, at small scale dimensions, the primary concerns are flow manipulation at low Reynolds number, precise pumping, active mixing. However, in order to deal with the mentioned issues, electroosmosis delivers a favorable approach to address these tasks. The fluid transport endorsed by electric field across the conduit is referred as electroosmosis. The submission of externally applied electric field nearby a solid–liquid edge may induce liquid flow owing to neighboring charged surface. This process generates EDL (electric double layer) at the solid–fluid edge by forming an ionic ambiance all around the charged species with assistance of boundary charges and its counter ions. Some noteworthy attempts high-lightening the significance of electroosmosis in microflows are reported in Refs.^1–5^. Burgreen and Nakache^6^ and Wang et al.^7^ debated the rational implications of electrokinetically driven pumping transport through microchannel. Mekheimer et al.^8^ have explored the impact of electric force on blood motion through peristaltic symmetric channel and informed that the submission of electric field in the flow direction strengthens blood flow. Concurrent effects of electric and magnetic field on ciliary transport of bio-fluid were examined by Munawar^9^. Prakash et al.^10^ have investigated electroosmosis regulated peristaltic motion of the Newtonian fluid enclosure with slip and radiation effects.

The intended thermoosmotic transport deals with the blood flow through micropump. On account of its rheology, the nature of blood running inside the electroosmotic pump is deemed as the Carreau fluid model^11,12^. But unfortunately, the traditional blood keeps deficient thermal conductivity. This inadequacy of conventional liquids has been conquered by introducing the concept of nanofluids. Nanofluid is prepared by the amalgamation of one or more types of metallic nanogranules with elevated thermal conductivity in conventional base fluid. The very first attempt introducing the idea of nanofluids was presented by Choi^13^. The ensuing fluid is identified as nanofluid that keeps higher heat transfer properties in contrast to conventional fluids. Due to its prospective application and ample uses, it has achieved immense attention of many researchers^14–16^. Over the course of time, researchers considered the hybridization of two distinct kind of metallic nanoparticles with base fluid to obtain bi-hybrid nanofluid with enhanced thermal characteristics than mono nanofluid. Since last decade, the investigation of hybrid nanofluid flow has become a mounting field of research. Some interesting applications of hybrid nature nanofluid are mentioned in threptic process^17^, drug supply^18^, micro heat exchangers^19^, lubricants^20^, cooling agents^21^ and thermal pumps^22^. The concept of ternary fluids that is the incorporation of trimetallic nanoparticles with base fluid has been recently established. Experimental attempts^23–25^ are given to report the significance and remarkable features of ternary nano fluids. Whereas a very few theoretical attempts have been presented to emphasize the promising and discerning role of trimetallic nanofluid in heat transfer augmentation. In this regard, a mathematical ternary fluid model elaborating enhanced heat transfer characteristics was given by Manjunatha et al.^26^. Elnaqeeb et al.^27^ explored the effects of stretching and suction on aqueous based trihybrid nanofluid. Recently, an entropic study of Williamson ternary nanofluid has been conducted by Munawar and Saleem^28^ and informed that radiated ternary fluid acquired enhanced heat transfer properties than bi/mono nanofluids.

At low Reynolds number, motile cilia play a considerable part in fluid manipulation and mixing. Cilia move in groups by performing backwards and forwards strikes in elliptical course to stimulate recurring waves named as metachronal waves. Some significant applications of fabricated cilia in the perspective of microfluidic handling, unidirectional flows, immediate pumping, and mixing are given^29–34^.

One of the important challenges in thermo-mechanical flows through microfluidic pump is thermal management. Recent trends in the development of bioengineered microelectronic devices require elevated thermal efficiency and effective cooling activity. The existence of substantial velocity and temperature differences in microchannel streams yield a huge heat indulges. Accumulation of unnecessary heat losses adversely influences device performance. Thus, to achieve an optimal thermo-mechanical design with proper thermal management, entropy formation requires to be diminished at most. Therefore, it is essential to find the ways that contribute to entropy minimization and flow optimization. Some interesting studies describing the entropic analysis in micro conduit are given^35–40^. A pioneering attempt regarding the entropy production in thermal system was contributed by Bejan^41^. Misra et al.^42^ and Munawar et al.^43^ presented optimum design of micropump and corrugated triangular cavity to reduce entropy formation. Basha et al.^44^ conducted entropic analysis of tangent hyperbolic nanofluid over a circular cylinder. Recently, entropic analysis of biological flows through ciliated channel was aided by Saleem and Munawar^45^ and Munawar et al.^46^.

The above literature review reveals that the mono and hybrid nanofluids flow in biological environments have been considered significantly. Nevertheless, the case of trihybrid nanofluids in physiological flows is still needed to be further explored, especially, in recent trends towards modern microfluidic appliances. To fill-up this gap, the current work aims to investigate the role of thermal radiation and applied elevated magnetic field in a forced convection motion of ternary nanofluid through an electroosmotic flexible pump. The ternary nanofluid is comprised of three distinct types of metallic nano-sized granules of titanium oxide, Silica and Aluminum dioxide blended with pure blood characterized by Carreau fluid model. The internal surface of pump is fabricated with uniform cilia layer with momentum slip boundary. The numerical solutions of the intended flow model are computed by shooting method and are validated by a comparison with a previously published study. A comparison between the thermal features of mono/hybrid/ternary nanofluids and traditional blood is presented. To the best of our knowledge such flow situation has never been considered earlier. The proposed study may provide a profound understanding in bioengineering models and drug supply.

## Mathematical formulation

### Problem definition

**Figure 1:**
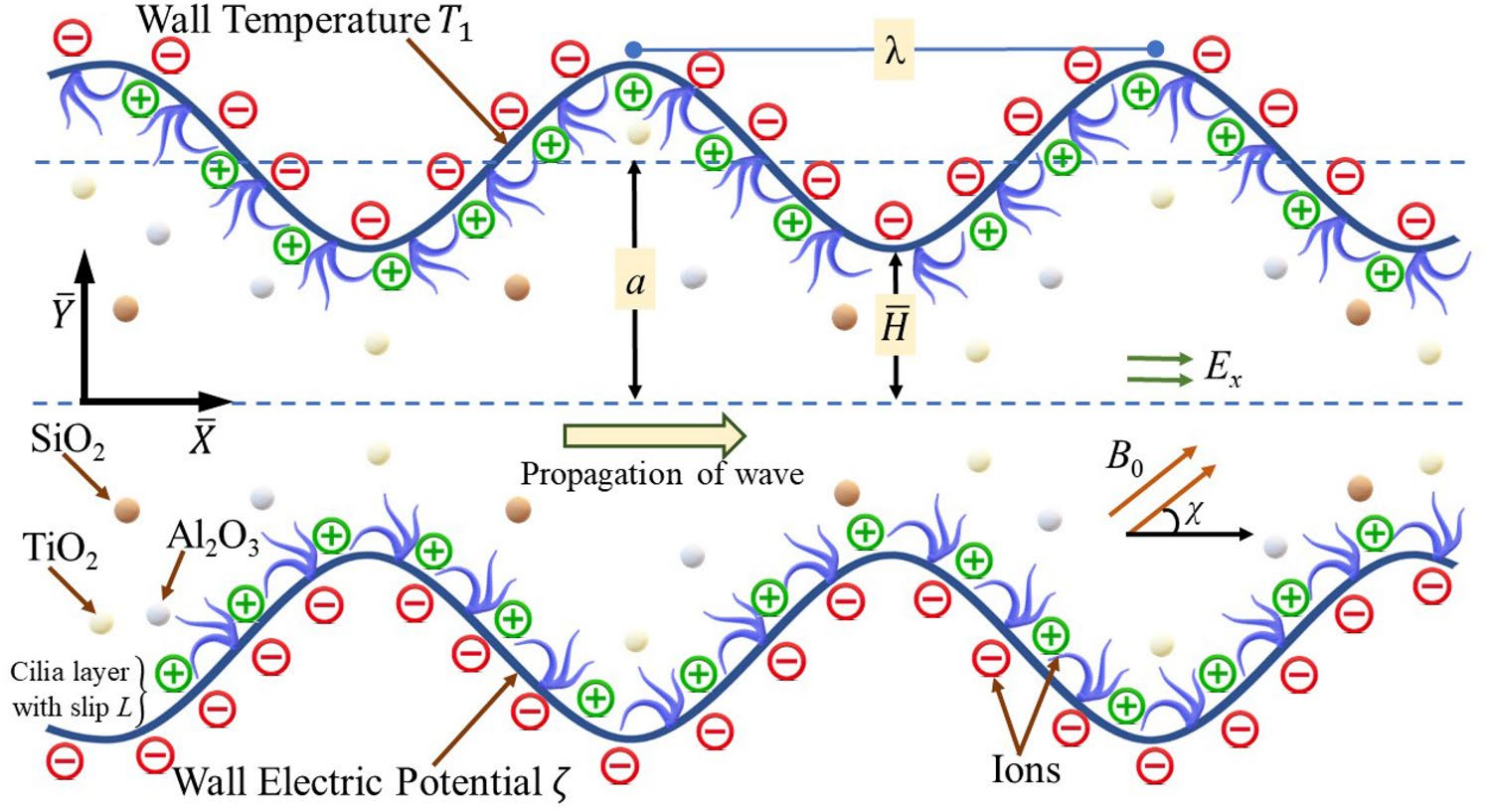
Geometrical model for cilia actuated electroosmotic transport of Carreau ternary nanofluid.

Consider a two-dimensional pumping motion of Carreau ternary fluid through an electroosmotic micropump in the presence of magnetic field applied at an angle *χ* from horizontal axis $\overline{X }$. The micropump is loaded with blood based trimetallic nanofluid obtained by the amalgamation of three distinct types of metallic nanogranules of Titanium dioxide (TiO_2_), silicon dioxide or silica (SiO_2_) and Alumina (Al_2_O_3_) with 1% volume proportion of each with pure blood (Carreau fluid model). The velocity partial slip at the surface of elastic walls of the pump is implemented with a slip length *λ*_1_. Moreover, the walls of pump are kept at a relatively higher temperature *T*_1_ and the pump also radiates the heat with flux ${\overline{q} }_{r}$. The pump is considered axially symmetric thus, the velocity and temperature gradients at the axis of pump can be assumed zero. Cilia whip in coordination to initiate a set of metachronal waves along the channel surface as portrayed in Fig. 1. The flow geometry is designed in a cartesian coordinate system in such a way that the wave transmission along $\overline{X }$-axis and the $\overline{Y }$-axis is normal to the flow. The entrenched cilia formation is described by the equation as^47^:

1 $$\overline{H }\left(\overline{X },\overline{t }\right)=a+a\varepsilon \text{cos}\left(\frac{2\pi }{\lambda }\left(\overline{X }-c\overline{t }\right)\right).$$

The cilia tips embrace in elliptical course and are horizontally positioned at (Sleigh^47^):

2 $$\overline{G }\left(\overline{X },\overline{t }\right)={X}_{0}+a\alpha \varepsilon \text{sin}\left(\frac{2\pi }{\lambda }\left(\overline{X }-c\overline{t }\right)\right),$$

where *a* stands for mean width of the ciliated channel, *ε* indicates the cilia length, *λ* denote the wavelength of metachronal wave, *α* represents measure of eccentricity of elliptic course, *X*_*0*_ is the location of liquid particle and $\overline{t }$ is used for time. The components of cilia velocity in horizontal and vertical directions can be obtained by differentiation of Eqs. (1) and (2) as

3 $${\overline{U} }_{0}={\left(\frac{\partial \overline{G} }{\partial \overline{t} }\right)}_{{X}_{0}}=\frac{-\left(\frac{2\pi }{\lambda }\right)a\varepsilon \alpha c\text{cos}\left(\frac{2\pi }{\lambda }\left(\overline{X }-c\overline{t }\right)\right)}{1-\left(\frac{2\pi }{\lambda }\right)a\varepsilon \alpha \text{cos}\left(\frac{2\pi }{\lambda }\left(\overline{X }-c\overline{t }\right)\right)},$$

4 $${\overline{V} }_{0}={\left(\frac{\partial \overline{H} }{\partial \overline{t} }\right)}_{{X}_{0}}=\frac{-\left(\frac{2\pi }{\lambda }\right)a\varepsilon \alpha c\text{sin}\left(\frac{2\pi }{\lambda }\left(\overline{X }-c\overline{t }\right)\right)}{1-\left(\frac{2\pi }{\lambda }\right)a\varepsilon \alpha \text{sin}\left(\frac{2\pi }{\lambda }\left(\overline{X }-c\overline{t }\right)\right)}.$$

For the Carreau trimetallic nanofluid, the extra stress tensor is communicated as^11,12^:

5 $$\overline{\mathbf{S}}=\left[{\mu }_{thnf}{\left(1+{\left(\Gamma \dot{\gamma }\right)}^{2}\right)}^{\frac{n-1}{2}}\right]{\mathbf{A}}_{1},$$

where $\overline{\dot{\gamma }}=\sqrt{\frac{1}{2}{\sum }_{i}{\sum }_{j}{\overline{\dot{\gamma }}}_{ij}{\dot{\gamma }}_{ji}}=\sqrt{\frac{1}{2}\Pi }$, where *n*,* μ*_*thnf*_*,* and Γ correspond to the power law index, the effective viscosity for Carreau trihybrid fluid, and the time relaxation parameter for ternary fluid. The second invariant strain tensor Π is specified as Π = trac(**A**_1_^2^), where **A**_1_ is the first Rivlin Erickson tensor. Hence

6 $$\overline{\dot{\gamma }}=\sqrt{2{\left(\frac{\partial \overline{U} }{\partial \overline{X} }\right)}^{2}+2{\left(\frac{\partial \overline{V} }{\partial \overline{Y} }\right)}^{2}+{\left(\frac{\partial \overline{U} }{\partial \overline{Y} }+\frac{\partial \overline{V} }{\partial \overline{X} }\right)}^{2}.}$$

The extra stress components are determined from Eq. (6) as

7 $${\overline{S} }_{\text{XX}}=2{\mu }_{thnf}\left[1+\left(\frac{n-1}{2}\right){\Gamma }^{2}{\dot{\gamma }}^{2}\right]\frac{\partial \overline{U} }{\partial \overline{X} },$$

8 $${\overline{S} }_{\text{XY}}={\mu }_{thnf}\left[1+\left(\frac{n-1}{2}\right){\Gamma }^{2}{\dot{\gamma }}^{2}\right]\left(\frac{\partial \overline{U} }{\partial \overline{Y} }+\frac{\partial \overline{V} }{\partial \overline{X} }\right),$$

9 $${\overline{S} }_{\text{YY}}={\mu }_{thnf} \left[1+\left(\frac{n-1}{2}\right){\Gamma }^{2}{\dot{\gamma }}^{2}\right]\frac{\partial \overline{V} }{\partial \overline{Y} }.$$

The Carreau model in Eq. (5) exhibits Newtonian fluid when *n* = 1 or Γ = 0.

### Governing equations in laboratory set up

Under the contemplation of above scenario, in laboratory frame, the conservation laws for flux, motion and heat in the presence of axially applied electric field **E** = (*E*_*x*_, 0) and uniform magnetic field **B** = (*B*_0_ sin χ, *B*_0_ cos χ) are noted as^48^

10 $$\frac{\partial \overline{U} }{\partial \overline{X} }+\frac{\partial \overline{V} }{\partial \overline{Y} }=0,$$

11 $$\begin{aligned}{\rho }_{thnf}\left(\frac{\partial \overline{U} }{\partial \overline{t} }+\overline{U }\frac{\partial \overline{U}}{\partial \overline{X} }+\overline{V }\frac{\partial \overline{U}}{\partial \overline{Y} }\right)&=-\frac{\partial \overline{P} }{\partial \overline{X} }+\frac{\partial {\overline{S} }_{\text{XX}}}{\partial \overline{X}}+\frac{\partial {\overline{S} }_{\text{XY}}}{\partial \overline{Y}}\\& \quad -{\sigma }_{thnf}{B}_{0}^{2}\text{cos}(\chi )\left(\overline{U}\text{cos }\chi -\overline{V}\text{sin }\chi \right)+{\rho }_{e}{E}_{x},\end{aligned}$$

12 $${\rho }_{thnf}\left(\frac{\partial \overline{V} }{\partial \overline{t} }+\overline{U }\frac{\partial \overline{V} }{\partial \overline{X} }+\overline{V }\frac{\partial \overline{V} }{\partial \overline{Y} }\right)=-\frac{\partial \overline{P} }{\partial \overline{Y} }+\frac{\partial {\overline{S} }_{\text{XY}}}{\partial \overline{X} }+\frac{\partial {\overline{S} }_{\text{YY}}}{\partial \overline{Y} }-{\sigma }_{thnf}{B}_{0}^{2}\text{sin}(\chi )\left(\overline{U}\text{cos }\chi -\overline{V}\text{sin }\chi \right),$$

13 $$\begin{aligned}{\left(\rho {C}_{P}\right)}_{thnf}\left(\frac{\partial \overline{T} }{\partial \overline{t} }+\overline{U }\frac{\partial \overline{T} }{\partial \overline{X} }+\overline{V }\frac{\partial \overline{T} }{\partial \overline{Y} }\right)&={k}_{thnf}\left(\frac{{\partial }^{2}\overline{T} }{\partial {\overline{X} }^{2}}+\frac{{\partial }^{2}\overline{T} }{\partial {\overline{Y} }^{2}}\right)+{\overline{S} }_{\text{XX}}\frac{\partial \overline{U} }{\partial \overline{X} }+{\overline{S} }_{\text{XY}}\left(\frac{\partial \overline{U} }{\partial \overline{Y} }+\frac{\partial \overline{V} }{\partial \overline{X} }\right)+{\overline{S} }_{\text{YY}}\frac{\partial \overline{V} }{\partial \overline{Y} }\\& \quad -\left(\frac{\partial {\overline{q} }_{r}}{\partial \overline{X} }+\frac{\partial {\overline{q} }_{r}}{\partial \overline{Y} }\right)+{\sigma }_{thnf}{E}_{x}^{2}+{\sigma }_{thnf}{B}_{0}^{2}\text{cos}(\chi )\left({\overline{U} }^{2}\text{cos}\chi -{\overline{V} }^{2}\text{sin}\chi \right),\end{aligned}$$

Subject to the boundary conditions

14 $$\left.\begin{array}{c}\overline{U }={\overline{U} }_{0}+{\lambda }_{1}{\mu }_{thnf}\left[1+\left(\frac{n-1}{2}\right){\Gamma }^{2}{\dot{\gamma }}^{2}\right]\left(\frac{\partial \overline{U} }{\partial \overline{Y} }+\frac{\partial \overline{V} }{\partial \overline{X} }\right), \overline{V }=0, \overline{T }={T}_{1} \text{ at} \overline{Y }=\overline{H },\\ \frac{\partial \overline{U} }{\partial \overline{Y} }=0, \frac{\partial \overline{T} }{\partial \overline{Y} }=0 \text{ at } \overline{Y }=0,\end{array}\right\} ,$$

where $\overline{U }$, $\overline{V }$, $\overline{P }$ and $\overline{T }$ represent the velocity components along horizontal and vertical direction, the ternary fluid pressure and temperature.

The magnitude of radiative heat flux is considered as negligible in the direction of fluid transport as compared to its orthogonal direction. Hence the representation of radiative heat flux under the Rosseland approximation is given as^18,19^:

15 $${\overline{q} }_{r}=\frac{-4{\sigma }^{*}}{3{K}^{* }}\frac{\partial {\overline{T} }^{4}}{\partial \overline{Y} },$$

in which K* and σ* correspond to the absorption coefficient and the Stefan-Boltzmann constant. The Taylor series expansion of $\overline{T }$^4^ about the temperature difference (∆T) in the presence of trivial temperature gradient in the micropump is noted as: $\overline{T}$^4^ ≅ 4 $\overline{T }$ (∆T)^3^ − 3(∆T)^4^. Therefore, Eq. (14) reduces to

16 $${\overline{q} }_{r}=\frac{-16{\sigma }^{*}{\left(\Delta \text{T}\right)}^{3}}{3{K}^{* }}\frac{\partial \overline{T} }{\partial \overline{Y} }.$$

By using the Poisson equation, the representation of electric potential distribution in fixed frame inside the micropump is conveyed as^8–10^

17 $$\frac{{\partial }^{2}\overline{\Phi } }{\partial {\overline{X} }^{2}}+\frac{{\partial }^{2}\overline{\Phi } }{\partial {\overline{Y} }^{2}}=-\frac{{\rho }_{e}}{\epsilon {\epsilon }_{0}},$$

where $\overline{\Phi  }$, *ϵ* and *ϵ*_0_ correspond to the electric potential, liquid’s relative permittivity and vacuum permittivity. For symmetric electrolyte binary liquid holding equal and opposite ions (positive $({\overline{n} }_{+})$ and negative ions $({\overline{n} }_{-})$), the net ionic distribution (*ρ*_*e*_) is expressed as^8^:

18 $${\rho }_{e}=ez\left({\overline{n} }_{+}-{\overline{n} }_{-}\right)=ez\left({n}_{o}{e}^{\frac{-ze\overline{\Phi }}{{k }_{b}{T}_{ave}}}-{n}_{o}{e}^{\frac{ze\overline{\Phi }}{{k }_{b}{T}_{ave}}}\right)=-2{n}_{o}ze\text{sinh}\left(\frac{ze\overline{\Phi }}{{k }_{b}{T}_{ave}}\right),$$

with *e, n*_*o*_, *k*_*b*_, *z* and *T*_*ave*_ respectively stand for the protonic charge, average concentration of positive and negative charges, the Boltzmann constant, the charge valence, and absolute temperature for medium. The supposition of homogeneous concentration of solid nanoparticles appears to be justifiable when there are insignificant concentration-gradients present in the ternary fluid and the appropriately small Peclet number.

It is further considered that the zeta-potential at pump surface (≤ 25 mV) is significantly small and thus the Debye-Hückel linearization^49^ turns to

19 $$\text{sinh}\left(\frac{ze\overline{\Phi }}{{k }_{b}{T}_{ave}}\right)\cong \frac{ze\overline{\Phi }}{{k }_{b}{T}_{ave}}.$$

On employing Eqs. (18) and (19) into Eq. (17), one gets

20 $$\frac{{\partial }^{2}\overline{\Phi } }{\partial {\overline{X} }^{2}}+\frac{{\partial }^{2}\overline{\Phi } }{\partial {\overline{Y} }^{2}}=\frac{2{n}_{o}{z}^{2}{e}^{2}}{{k}_{b}{T}_{ave}\epsilon {\epsilon }_{o}}\overline{\Phi  }.$$

### Thermophysical characteristics of ternary fluid (TiO_2_–SiO_2_–Al_2_O_3_/blood)

It is considered that the blood based ternary fluid is diluted colloidal suspension of trimetallic nanogranules of Titanium dioxide (TiO_2_), silicon dioxide (SiO_2_) and Aluminium oxide (Al_2_O_3_) assorted thoroughly in pure blood. Titanium dioxide possesses various applications in medicines, chemical and textile engineering due to its coolant and photo catalytic properties. Whereas, silicon dioxide nanogranules are commonly used in drug delivery owing to its nontoxic nature and stability. Aluminium oxide is ideal for pharmaceuticals and materials manufacturing industries owing to its corrosion and thermal resistance properties. The numerical values of thermophysical properties for the considered nanoparticles and base-fluid are reported in Table 1 at the reference temperature of 25 °C. The mathematical expressions that unfolding the thermophysical features of trimetallic nanofluids are specified as^26,27^:

21 $${\rho }_{thnf}=\left(1-{\phi }_{1}\right)\left[\left(1-{\phi }_{2}\right)\left\{\left(1-{\phi }_{3}\right){\rho }_{f}+{{\phi }_{3}\rho }_{3}\right\}+{\phi }_{2}{\rho }_{2}\right]{+\phi }_{1}{\rho }_{1},$$

22 $${\left(\rho {C}_{p}\right)}_{thnf}=\left(1-{\phi }_{1}-{\phi }_{2}-{\phi }_{3}\right){\left(\rho {C}_{p}\right)}_{f}+{{\phi }_{1}\left(\rho {C}_{p}\right)}_{1}+{{\phi }_{2}\left(\rho {C}_{p}\right)}_{2}+{{\phi }_{3}\left(\rho {C}_{p}\right)}_{3},$$

23 $$\frac{{\sigma }_{thnf}}{{\sigma }_{f}}=\frac{\left(1+2{\phi }_{1}\right){\sigma }_{1}+\left(1-2{\phi }_{1}\right){\sigma }_{hnf}}{\left(1-{\phi }_{1}\right){\sigma }_{1}+\left(1+{\phi }_{1}\right){\sigma }_{hnf}},$$

with $$\frac{{\sigma }_{hnf}}{{\sigma }_{nf}}=\frac{\left(1+2{\phi }_{2}\right){\sigma }_{2}+\left(1-2{\phi }_{2}\right){\sigma }_{nf}}{\left(1-{\phi }_{2}\right){\sigma }_{2}+\left(1+{\phi }_{2}\right){\sigma }_{nf}}$$ and $$\frac{{\sigma }_{nf}}{{\sigma }_{f}}=\frac{\left(1+2{\phi }_{3}\right){\sigma }_{3}+\left(1-2{\phi }_{3}\right){\sigma }_{f}}{\left(1-{\phi }_{3}\right){\sigma }_{3}+\left(1+{\phi }_{3}\right){\sigma }_{f}}$$,

24 $$\frac{{k}_{thnf}}{{k}_{hnf}}=\frac{{k}_{1}+2{k}_{hnf}-2{\phi }_{1}\left({{k}_{hnf}-k}_{1}\right)}{{k}_{1}+2{k}_{hnf}+{\phi }_{1}\left({{k}_{hnf}-k}_{1}\right)},$$

with $$\frac{{k}_{hnf}}{{k}_{nf}}=\frac{{k}_{2}+2{k}_{nf}-2{\phi }_{2}\left({{k}_{nf}-k}_{2}\right)}{{k}_{2}+2{k}_{nf}+{\phi }_{2}\left({{k}_{nf}-k}_{2}\right)}$$ and $$\frac{{k}_{nf}}{{k}_{f}}=\frac{{k}_{3}+2{k}_{f}-2{\phi }_{3}\left({{k}_{f}-k}_{3}\right)}{{k}_{3}+2{k}_{f}+{\phi }_{3}\left({{k}_{f}-k}_{3}\right)}$$,

25 $$\frac{{\mu }_{thnf}}{{\mu }_{f}}=\frac{1}{{\left(1-{\phi }_{1}\right)}^{5/2}{\left(1-{\phi }_{2}\right)}^{5/2}{\left(1-{\phi }_{3}\right)}^{5/2}},$$

in which *ϕ* denotes the volume fraction of metallic nanogranules, *ρ* denotes the density, *ρC*_*p*_ stands for heat capacity, *σ* the electrical conductivity, *k* is the thermal conductivity and *µ* holds for viscosity . The numeric subscripts 1, 2 and 3 correspond to the characteristics associated with solid nanogranules of TiO2, SiO_2_, and Al_2_O_3_. The alphabetical index *thnf*, *hnf*, *nf* and *f* respectively denote the characteristics related with ternary, binary, mono nanofluid and blood.

**Table 1:** Thermophysical features of the blood based ternary nanofluid^26–28^.

Physical entities	Base fluid	Solid nanogranules		
	(Blood)	TiO_2_	SiO_2_	Al_2_O_3_
*ρ* (kg m^−3^)	1063	4250	2200	3970
*σ* (1/Ωm)	0.8	2.4 × 10^6^	3.5 × 10^6^	36.9 × 10^6^
*C*_*p*_ (J K^−1^ kg^−1^)	3594	686.2	754	765
*k* (W m^−1^ K^−1^)	0.492	8.9538	1.4013	40

### Governing equations in wave frame

The inter conversion relationship between the fixed frame and moving frame are defined by the following mathematical equations^5–7^:

26 $$\overline{x }=\overline{X }-c\overline{t }, \overline{y }=\overline{Y }, \overline{p }\left(x,y\right)=\overline{P }\left(\overline{X },\overline{Y },\overline{t }\right), \overline{u }\left(x,y\right)=\overline{U }\left(\overline{X },\overline{Y },\overline{t }\right)-c, \overline{v }\left(x,y\right)=\overline{V }\left(\overline{X },\overline{Y },\overline{t }\right).$$

Afterwards, Introducing the following non-dimensional quantities to nondimensionalize the governing equations^36,48^:

27 $$\left.\begin{array}{c}x=\frac{\overline{x}}{\lambda  }, y=\frac{\overline{y}}{a }, t=\frac{c\overline{t}}{a }, \beta =\frac{a}{\lambda }, u=\frac{\overline{u}}{c }, v=\frac{\overline{v}}{\beta c }, p=\frac{\overline{p}{a }^{2}}{{\mu }_{f}c\lambda } \\ \theta =\frac{\overline{T }-{T}_{0}}{{T}_{1}-{T}_{0}}, S=\frac{\overline{S}a}{{\mu  }_{f}c},\Phi =\frac{\overline{\Phi }}{\zeta  }, H=\frac{\overline{H}}{a }, \text{Re}=\frac{{\rho }_{f}ac}{{\mu }_{f}} \end{array}\right\}.$$

Presenting the stream function (Ψ) in the form

28 $$u=\frac{\partial\Psi }{\partial y}, v=-\frac{\partial\Psi }{\partial x}.$$

After using Eqs. (26)–(28) into Eqs. (10)–(13) and (20) and applying long wavelength (*β* ≪ 1) and the small Reynolds number (Re ≈ 0) approximations, the dimensionless forms of the governing equations are reported as:

29 $$\frac{\partial p}{\partial x}=\frac{{\mu }_{thnf}}{{\mu }_{f}}\left[\frac{{\partial }^{3}\Psi }{\partial {y}^{3}}+\frac{n-1}{2}{\text{We}}^{2}\frac{\partial }{\partial y}{\left(\frac{{\partial }^{2}\Psi }{\partial {y}^{2}}\right)}^{3}\right]-{\frac{{\sigma }_{thnf}}{{\sigma }_{f}}\text{Ha}}^{2}{\text{cos}}^{2}\chi \left(\frac{\partial\Psi }{\partial y}+1\right)+{U}_{HS}\frac{{\partial }^{2}\Phi }{\partial {y}^{2}},$$

30 $$\frac{\partial p}{\partial y}=0,$$

31 $$\left(\frac{{k}_{thnf}}{{k}_{f}}+{R}_{n}\right)\frac{{\partial }^{2}\theta }{\partial {y}^{2}}+\frac{{\mu }_{thnf}}{{\mu }_{f}}\text{EcPr}\left[{\left(\frac{{\partial }^{2}\Psi }{\partial {y}^{2}}\right)}^{2}+\frac{n-1}{2}{\text{We}}^{2}{\left(\frac{{\partial }^{2}\Psi }{\partial {y}^{2}}\right)}^{4}\right]+\frac{{\sigma }_{thnf}}{{\sigma }_{f}}\left[{S}_{p}+{\text{PrEcHa}}^{2}{\text{cos}}^{2}\chi {\left(\frac{\partial\Psi }{\partial y}+1\right)}^{2}\right]=0,$$

32 $$\frac{{\partial }^{2}\Phi }{\partial {y}^{2}}={\text{K}}^{2}\Phi .$$

Cross-differentiating Eqs. (29) and (30) gives

33 $$\frac{{\mu }_{thnf}}{{\mu }_{f}}\left[\frac{{\partial }^{4}\Psi }{\partial {y}^{4}}+\frac{n-1}{2}{\text{We}}^{2}\frac{{\partial }^{2}}{\partial {y}^{2}}{\left(\frac{{\partial }^{2}\Psi }{\partial {y}^{2}}\right)}^{3}\right]+{\frac{{\sigma }_{thnf}}{{\sigma }_{f}}\text{Ha}}^{2}{\text{cos}}^{2}\chi \frac{{\partial }^{2}\Psi }{\partial {y}^{2}}+{U}_{HS}\frac{{\partial }^{3}\Phi }{\partial {y}^{3}}=0.$$

Correspondingly, the boundary conditions are stated as

34 $$\left.\begin{array}{c}\Psi =0, \frac{{\partial }^{2}\Psi }{\partial {y}^{2}}=0, \frac{\partial \theta }{\partial y}=0, \frac{\partial\Phi }{\partial y}=0,\text{ at }y=0,\\\Psi =\text{F}, \theta =\Phi =1\text{ at }y=H,\\ \frac{\partial\Psi }{\partial y}+L\frac{{\mu }_{thnf}}{{\mu }_{f}}\left[\frac{{\partial }^{2}\Psi }{\partial {y}^{2}}+\frac{n-1}{2}{\text{We}}^{2}{\left(\frac{{\partial }^{2}\Psi }{\partial {y}^{2}}\right)}^{3}\right]=-1-\frac{2\pi \alpha \varepsilon \beta \text{cos}(2\pi x)}{1-2\pi \alpha \varepsilon \beta \text{cos}\left(2\pi x\right)}\text{ at }y=H\end{array}\right\}.$$

The non-dimensional quantities arising in the governing equations such as the Hartmann number (Ha), the Weissenberg number (We), the Prandtl number (Pr), the Eckert number (Ec), the Helmholtz–Smoluchowski velocity (*U*_*HS*_), the Knudsen number (*L*), the thermal radiation number (*R*_*n*_), the Joule heating parameter (*S*_*p*_) and the electroosmosis number (K) are expressed as

35 $$\left.\begin{array}{c}\text{Ha}=\sqrt{\frac{{\sigma }_{f}}{{\mu }_{f}}}{B}_{0}a, \text{We}=\frac{\Gamma c}{a}, \text{Pr}=\frac{{\mu }_{f}{C}_{P}}{{k}_{f}}, \text{Ec}=\frac{{c}^{2}}{{C}_{P}\Delta T}, {U}_{HS}=\frac{-{E}_{x}\epsilon {\epsilon }_{o}\zeta }{c{\mu }_{f}}\\ L=\frac{{\lambda }_{1}}{a}\sqrt{\frac{ca}{{\nu }_{f}}}, {R}_{n}=\frac{16{\sigma }^{*}{\left(\Delta T\right)}^{3}}{3{\mu }_{f}{C}_{P}{K}^{* }}, {S}_{p}=\frac{{\sigma }_{f}{E}_{x}^{2}{a}^{2}}{\Delta \overline{T}{k }_{f}}, \text{K}=aze\sqrt{\frac{2{n}_{o}}{{k}_{b}{T}_{ave}\epsilon {\epsilon }_{o}}}\end{array}\right\}.$$

The transformation between the volume flow rates in the laboratory (*Q*) frame and the wave (*F*) frame is stated by the following equation:

36 $$Q=1+F, \text{where }F={\int }_{0}^{H}\left(\frac{\partial\Psi }{\partial y}\right)dy.$$

The Nusselt number (Nu) is defined as:

37 $$\text{Nu}=-\frac{\partial H}{\partial x}{\left.\frac{\partial \theta }{\partial y}\right|}_{y=H}.$$

## Solution of the problem

Equation (32) along with the boundary conditions BCS (34) is a simple two-point second order BVP that can be solved exactly. The exact solution is calculated as

38 $$\Phi ={e}^{\left(H-y\right)\text{K}}\left(\frac{1+{e}^{2\text{K}y}}{1+{e}^{2\text{KH}}}\right).$$

However, Eqs. (31) and (33) along with BCS (34) form a system of forth order coupled nonlinear boundary value problem BVP. Such equations can easily be solved numerically by using numerical package “NDSolve” of Mathematica. While computing the solution, the built-in shooting method utility of “NDSolve” is called-on. In shooting method, a BVP is first adapted as an initial value problem IVP. Then this IVP is solved for guessed initial conditions until the solution is obtained that satisfies the boundary conditions on the other end.

**Figure 2:**
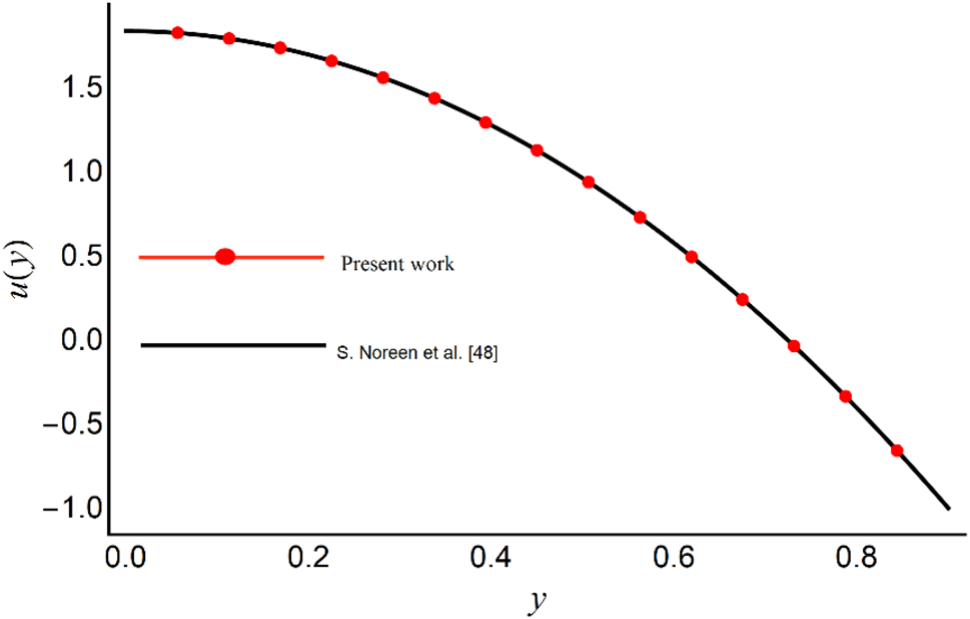
Comparison of the axial velocity profile of present work with the velocity distribution presented by S, Noreen et al.^48^.

**Figure 3:**
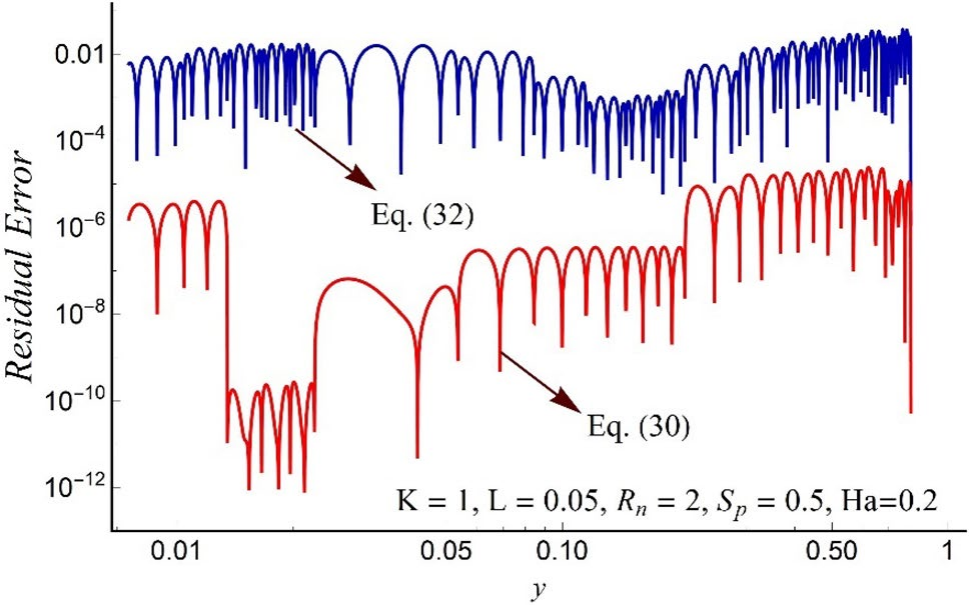
The residual error for the dimensionless governing Eqs. (31) and (33).

To validate our code and the numerical solution, we compare our result at limiting case with an analytic series solution provided in Ref.^48^. For this purpose, we assume *ϕ* → 0, *χ* → 0, Ha → 0, *S*_*p*_ → 0 and *L* → 0 and solved the limiting problem numerically. A comparison of this numerical solution with the perturbation series solution reported by Ref.^48^ is provided in Fig. 2 for both velocity profiles. The figure shows a good match between the perturbation series solution and the numerical shooting method. The residual errors for Eqs. (31) and (33) are sketched in Fig. 3. The figure shows that the errors are with-in permissible range which indicates the good accuracy of our results. Thus, the authenticity of the present numerical solution is verified.

## Entropy analysis

In the ciliated electroosmotic pump, the primary factors to yield entropy are heat transfer effects due to thermal radiation and convection, fluid friction, the Joule heating and electric potential. Therefore, the entropy generation equation for the intended flow in the regime of second law of thermodynamics is expressed as^41–43^:

39 $$ \begin{aligned}   S_{{gen}}^{{'''}}  =  & \frac{1}{{T_{0}^{2} }}\left[ {k_{{thnf}} \left( {\nabla \bar{T}} \right)^{2}  + \frac{{16\sigma ^{{\text{*}}} \left( {T_{1}  - T_{0} } \right)^{3} }}{{3K^{{{\text{*~}}}} }}\left( {\nabla \bar{T}} \right)^{2} } \right] \\     &  + \frac{{\mu _{{thnf}} }}{{T_{0} }}\left[ {{\mathbf{S}} \cdot \nabla {\mathbf{V}}} \right] + \frac{{\sigma _{{thnf}} }}{{T_{0} }}\left[ {B_{0}^{2} \cos \chi \left( {\bar{U}^{{~2}} \cos \chi  - \bar{V}^{{~2}} \sin \chi } \right) + E_{x}^{2} } \right], \\  \end{aligned}  $$

on execution of Eqs. (26) and (27) in Eq. (39) and dividing with characteristic entropy *S*_*G*0_ provides the total entropy number *N*_*S*_ as

40 $$  \begin{aligned}   S_{{gen}}^{{'''}}  =  & \frac{1}{{T_{0}^{2} }}\left[ {k_{{thnf}} \left( {\nabla \bar{T}} \right)^{2}  + \frac{{16\sigma ^{{\text{*}}} \left( {T_{1}  - T_{0} } \right)^{3} }}{{3K^{{{\text{*~}}}} }}\left( {\nabla \bar{T}} \right)^{2} } \right] \\     &  + \frac{{\mu _{{thnf}} }}{{T_{0} }}\left[ {{\mathbf{S}} \cdot \nabla {\mathbf{V}}} \right] + \frac{{\sigma _{{thnf}} }}{{T_{0} }}\left[ {B_{0}^{2} \cos \chi \left( {\overline{U} ^{2} \cos \chi  - \overline{V} ^{2} \sin \chi } \right) + E_{x}^{2} } \right], \\  \end{aligned}  $$

In Eq. (40) the very first part namely ${N}_{H}$ defines the heat transfer irreversibility because of heat transfer effects due to the two modes namely radiation and convection, the second term that is ${N}_{F}$ arises due fluid friction irreversibility, whereas the last term named as ${N}_{J}$ describes the entropy generation owing to the magnetic field and electric current. Moreover, dimensionless number *τ* (= ∆*T*/*T*_0_) is kept equal to one.

The Bejan number is named after Adrian Bejan and is specified as

41 $$\text{Be}=\frac{{N}_{H}}{{{N}_{H}+N}_{F}+{N}_{J}}.$$

In the regime of thermodynamics, the Bejan number belongs to [0, 1] and distinguishes the heat transfer irreversibility effect and the system’s total irreversibilities owing to other causes. The values of Be greater than 0.5 describes the consequential role of heat transfer irreversibility over the total irreversibility of the system. Also, the values of Be lower than 0.5 declare ascendancy of the system’s combined irreversibility attributable to the other causes like fluid friction and magnetic field and electric potential.

## Numerical findings and interpretation

**Figure 4:**
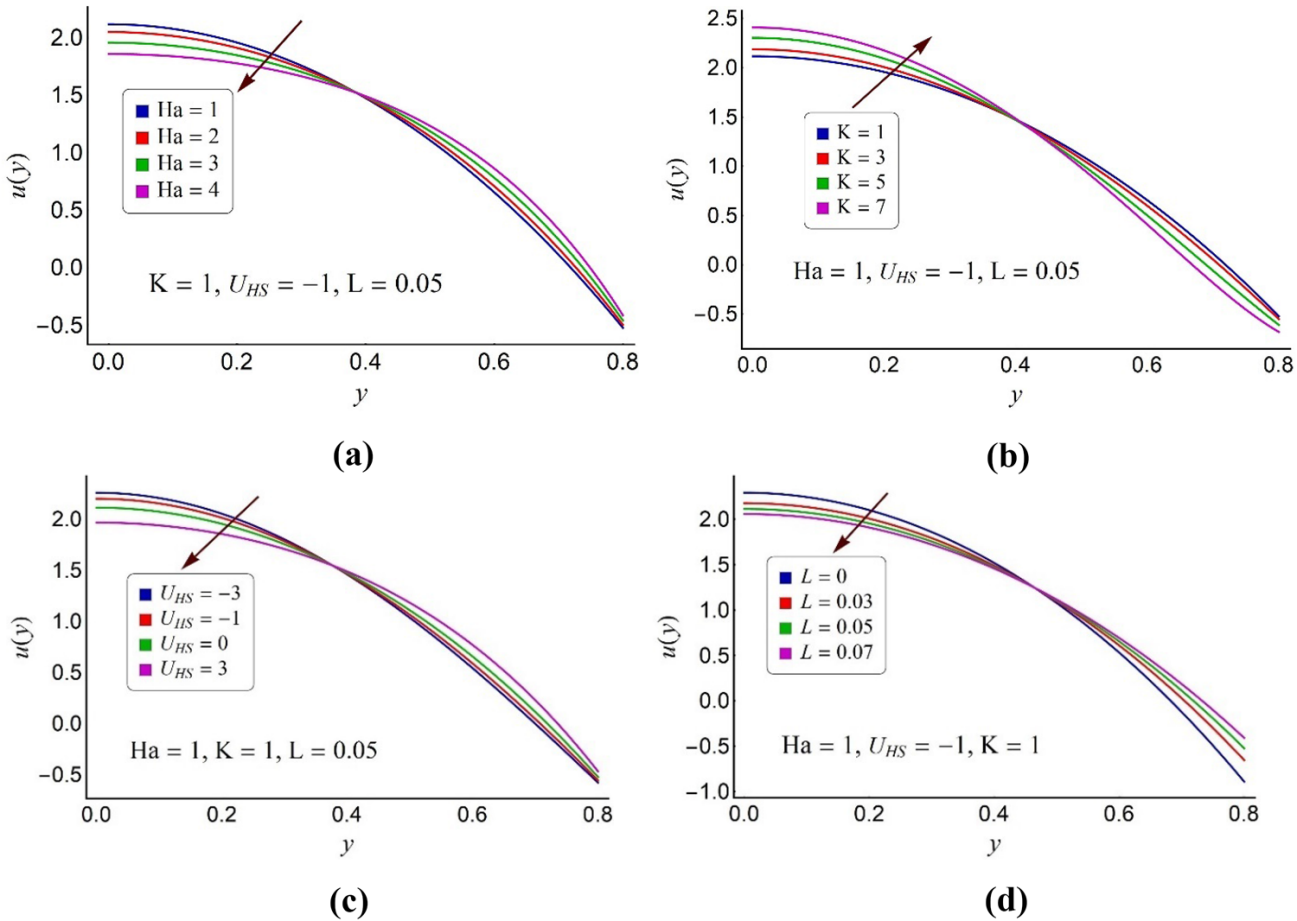
Alterations in velocity distribution of ternary fluid for various values of (**a**) Hartmann number Ha, (**b**) electroosmotic parameter K, (**c**) Helmholtz–Smoluchowski velocity *U*_*HS*_, (**d**) momentum slip parameter *L*.

**Figure 5:**
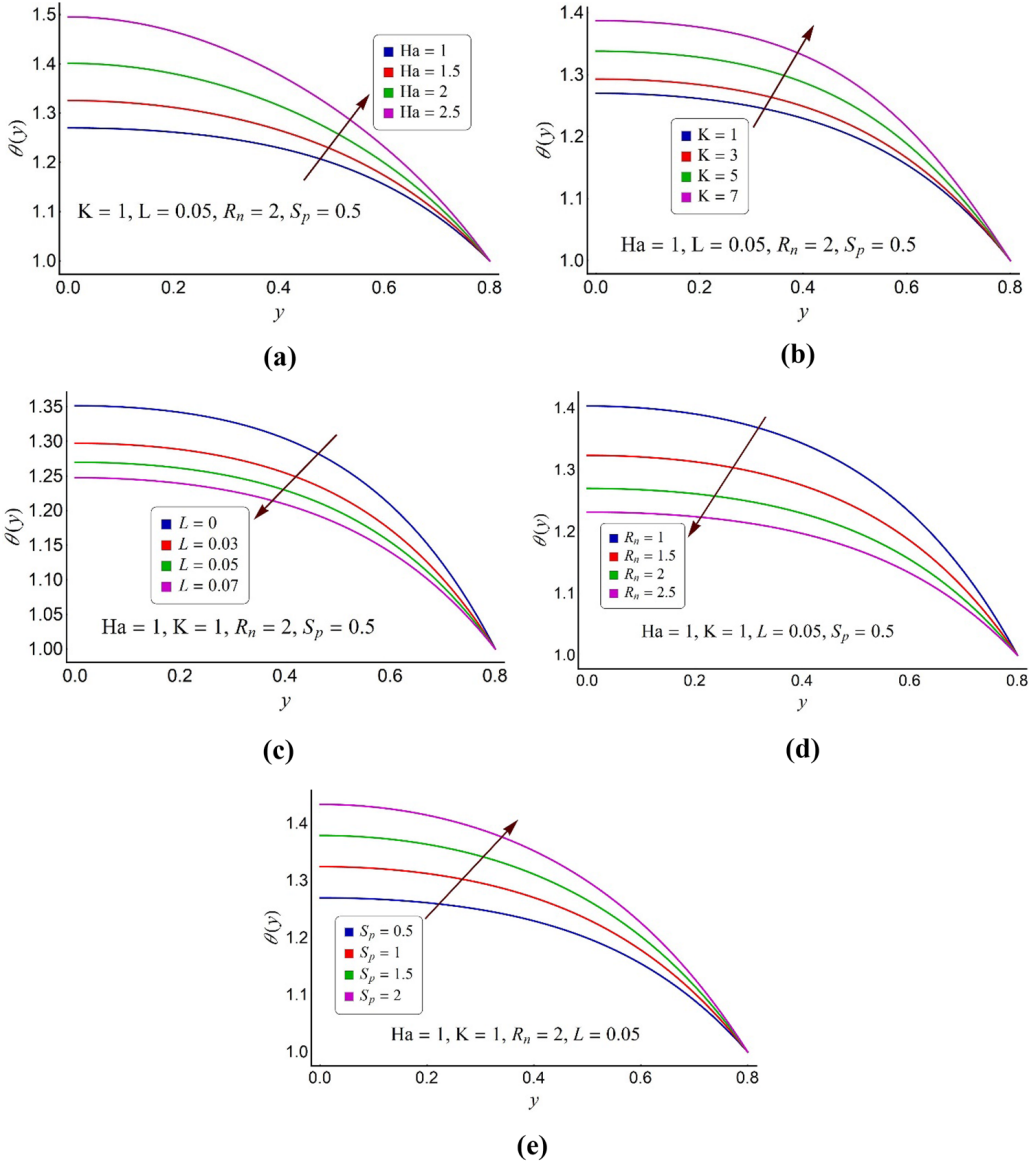
Alterations in temperature distribution of ternary fluid for various values of (**a**) Hartmann number Ha, (**b**) electroosmotic parameter K, (**c**) momentum slip parameter *L*, (**d**) thermal radiation number, (**e**) ohmic heating parameter *S*_*p*_.

**Figure 6:**
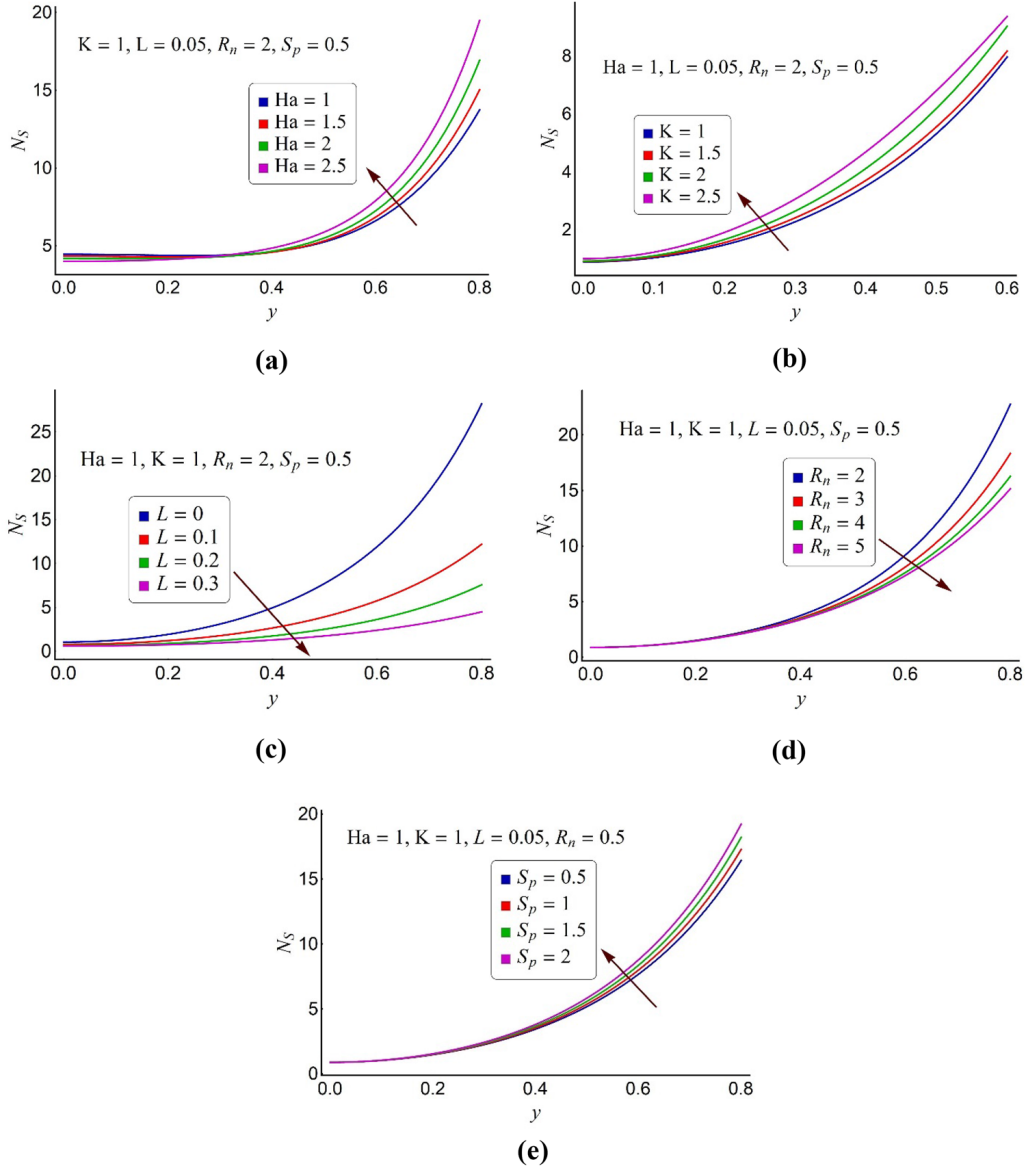
Alterations in the entropy formation number of ternary fluid for various values of (**a**) Hartmann number Ha, (**b**) electroosmotic parameter K, (**c**) momentum slip parameter *L*, (**d**) thermal radiation number *R*_*n*_, (**e**) ohmic heating parameter *S*_*p*_.

**Figure 7:**
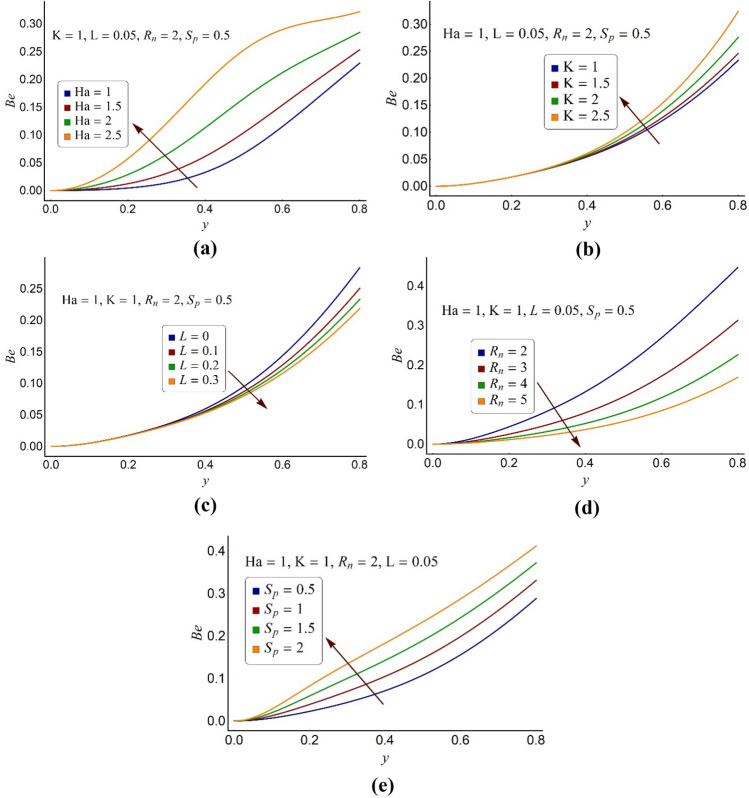
Alterations in the Bejan number profile of ternary fluid for various values of (**a**) Hartmann number Ha, (**b**) electroosmotic parameter K, (**c**) momentum slip parameter *L*, (**d**) thermal radiation number *R*_*n*_, (**e**) ohmic heating parameter *S*_*p*_.

**Figure 8:**
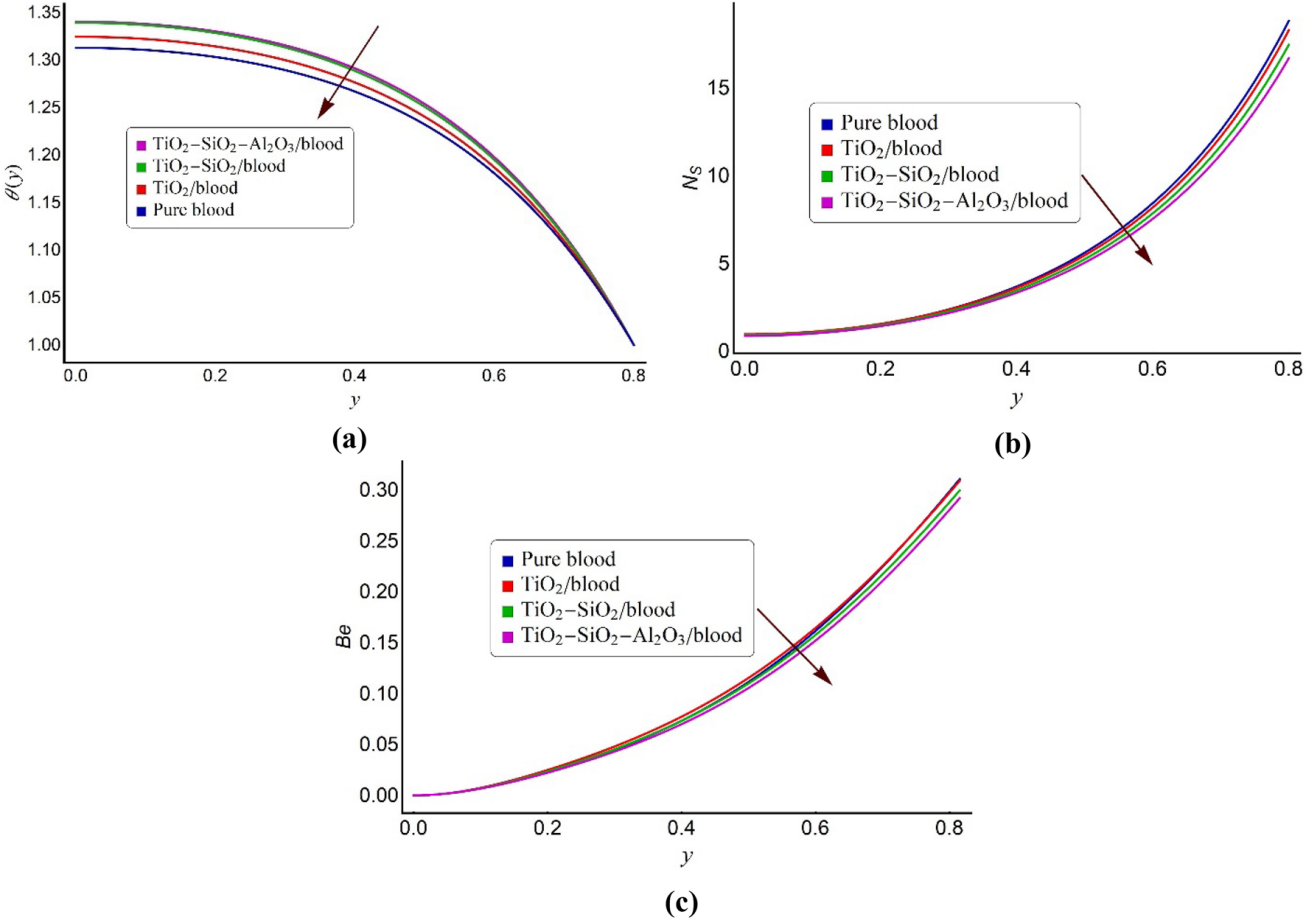
A comparative glimpse of tri-metallic/bi-metallic/mono-metallic nanofluid and conventional blood model for (**a**) the temperature distribution, (**b**) the entropy formation number, (**c**) the Bejan number profile.

**Figure 9:**
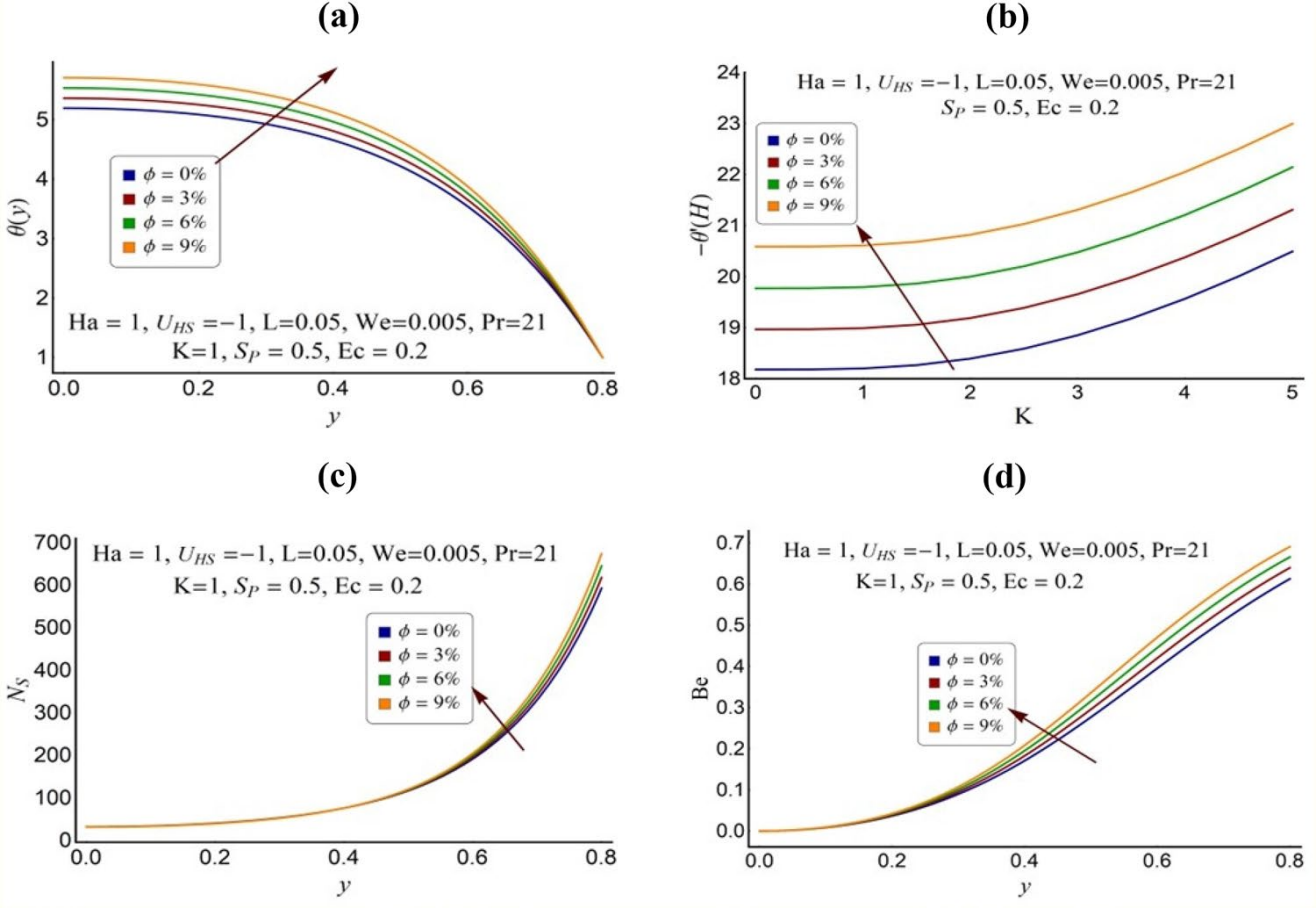
Effect of nanoparticles volume fraction (**a**) on the temperature profile (**b**) on the heat transfer rate (**c**) on the total entropy number and (**d**) on the Bejan number.

**Figure 10:**
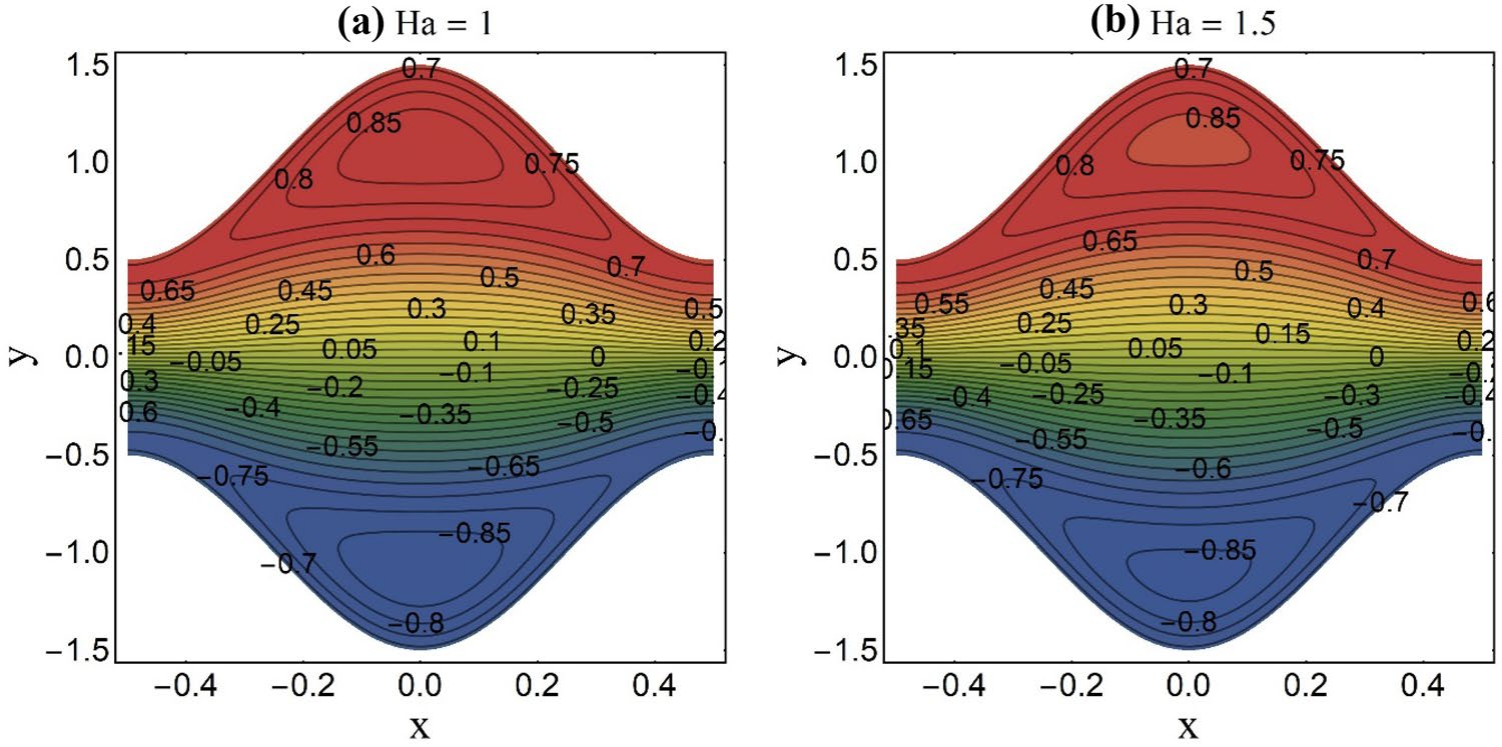
Streamlines pattern at fixed K = 2 and *U*_*HS*_ =  − 1.

**Figure 11:**
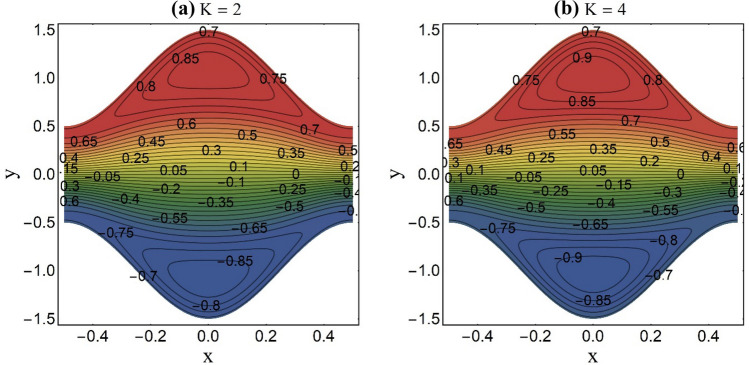
Streamlines pattern at fixed Ha = 1 and *U*_*HS*_ =  − 1.

**Figure 12:**
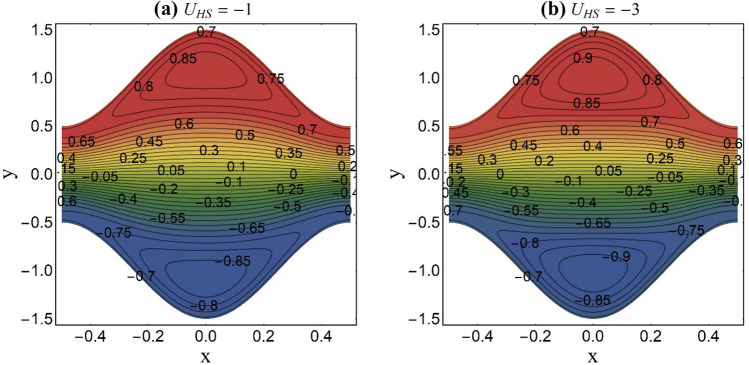
Streamlines pattern at fixed K = 2 and Ha = 1.

This section is focused for the interpretation of numerical simulation of the computational results for the velocity distribution (*u*), the streamline pattern (Ψ), the temperature profile (*θ*), the entropy formation number (*N*_*S*_) and the Bejan number (Be) for the ternary nanofluid. Several graphs for sundry values of the pertinent parameters of concern are delivered by Figs. 4, 5, 6, 7, 8, 9, 10, 11 and 12. In the entire assessment the overall dissipated proportion of metallic nanogranules, *ϕ* (= *ϕ*_1_ + *ϕ*_2_ + *ϕ*_3_) dispersed in the conventional blood is assumed as 3%. Thus, the blood based ternary nanofluid is supposed to be prepared by mixing 1% concentration of every type of solid nanogranules like titanium dioxide, silicon dioxide and Alumina in the Carreau fluid. Since the present parametric analysis is conducted for applicable parameters of interest, therefore some of the parameters are kept fixed such as the values of the Eckert number, the cilia length parameter and the pawer law index are select as 0.2, the Prandtl number is chosen as 1, The Weissenberg number is 0.01, inclination angle for the magnetic field is π/3, wave number and the value for the measure of eccentricity parameter are selected as 0.2.

### Flow characteristics of the ternary fluid

To study the alternations in the velocity profile of the ternary nanofluid for altered values of the magnetic number (Ha), the electroosmotic parameter (K), the Helmholtz–Smoluchowski velocity (*U*_*HS*_) and the momentum slip parameter (*L*), Fig. 4a–d is presented. Figure 4a suggests that the high values of the magnetic number (Ha) hinder the fluid stream in the proximity of pump deep part. Whereas the raised values of the Ha assist the fluid stream around the peripheral region of the micro pump. In fact, the association of transverse electromotive force with the magnetic field proposes a diminishing impression of Ha on ternary fluid stream in the pump core section. However, in order to balance the same flow rate in stretchy corrugated pump, an entire contrary conduct of velocity pattern is reported near the pump surface. Figure 4b illustrates the impact of electric double layer on ternary fluid velocity profile. The inverse of electric double layer (EDL) thickness is identified as electroosmotic parameter (K). Hence the strong EDL effects cause an intensification in fluid flow near the pump peripheral zone and a deterioration near the pump center. The applied external electric field has a linear relationship with the Helmholtz–Smoluchowski velocity (*U*_*HS*_). The positive values of *U*_*HS*_ represent the application of the electric field in the opposite direction of the ternary fluid stream. The counter positive values of *U*_*HS*_ lead to the submission of the electric field in the flow direction. Furthermore, the value of* U*_*HS*_ equal to zero implies the absence of electric field. Thus, Fig. 4c elaborates that in the vicinity of the pump core part, the implication of electric field in flow direction assists the fluid motion and when applied in the reverse direction of the flow, it impedes the fluid motion. Figure 4d suggests that the high values of momentum slip parameter (*L*) causes an acceleration in fluid flow around the pump surface region. But near the pump core section, this conduct of ternary fluid motion is entirely reverse.

### Temperature profile of the trimetallic nanofluid

Figure 5a–e portray the alternations in temperature profile of the trimetallic nanofluid for sundry values of some relevant parameters of concern like magnetic number (Ha), the electroosmotic parameter (K), momentum slip parameter (L), the radiation parameter (*R*_*n*_) and the Joule heating parameter (*S*_*p*_), respectively. Figure 5a shows that the lofty values of the Hartmann number (Ha) contribute to ternary fluid temperature enhancement owing to the sizeable convection effects. This rise in temperature is more noticeable around the pump core zone compared to the pump surface. Figure 5b describes that the raised values of electroosmotic number (K) cause an augmentation in the ternary fluid temperature throughout the pump region. But this rise is more pronounced around the pump core zone since the weak EDL effects aid to convection near the pump core portion. Figure 5c infers that the ternary fluid temperature can be lowered by integrating the momentum slip effects on the pump surface. An increment in the thermal radiation number (*R*_*n*_) induces a substantial deterioration in the ternary fluid temperature as seen through Fig. 5d. This act is concluded as the nonlinear relationship of heat radiation effects with thermal conduction triggers a perceptible deceleration in the ternary fluid temperature. Consequently, the radiated ternary fluid can receive as desirable factor in cooling process compared to the conventional mono/hybrid nanofluids. Figure 5e determines that the ternary fluid temperature augments for the large values of the Joule heating parameter (*S*_*p*_) owing to the internal heat creation by electric current.

### The entropic analysis

Measure of randomness and chaos in the system is identified as entropy of the system. In order to attain optimized efficiency of the flow system, heat indulges during an irreversible process should be lessened. In order to conduct second law analysis of the trimetallic nanofluid for various values of magnetic number (Ha), the electroosmotic parameter (K), momentum slip parameter (L), the thermal radiation parameter (*R*_*n*_) and the Joule heating parameter (*S*_*p*_), Fig. 6a–e is displayed. Figure 6a establishes that the elevated values of the magnetic number (Ha) contribute to an augmentation in entropy production around the pump surface. But near the pump core part, the variations in entropy generation number are inconsequential. It can be viewed through Fig. 6b that entropy in the electroosmotic pump rises when higher values of the electroosmotic parameter (weak EDL effects) are selected. Furthermore, this upsurge in entropy generation number gradually rises as it transfers from the pump center to the peripheral area. Figure 6c reports that entropy generation in the pump can be curbed by incorporating the velocity slip situation at the pump wall. It also shows that the entropy generation number is maximum for no-slip effects (*L* = 0), and it considerably reduces when taking large values of *L*. Figure 6d convenes that around the pump peripheral region, entropy generation in the micro pump decelerates when the raised values of the thermal radiation parameter (*R*_*n*_) are considered. This may compel that the thermally radiated ternary nanofluid can optimize the thermal efficiency of the flow system. To examine the influence of the ohmic heating number (*S*_*p*_) on the total entropy generation number of ternary fluid, Fig. 6e is plotted. This figure declares that entropy generation inside the wavy pump intensifies for large values of *S*_*p*_.

### Effect on the Bejan number

The Bejan number conveys a profound perception about the discrimination in various kinds of irreversibilities. In the regime of thermodynamics, the Bejan number is the ratio of heat transfer irreversibility (convection and radiation) to the system’s overall irreversibility owing to the other causes like fluid friction, conduction, and the joule heating etc. The values of the Bejan number alter from zero to one inclusive. Its value lower than 0.5 demonstrates the leading effect of fluid friction and other irreversibilities over the heat transfer irreversibility. While its value greater than 0.5 represents the ascendancy of heat transfer irreversibility over the system’s total irreversibility due to other causes. Figure 7a infers that a substantial increase in the Bejan number profile of the ternary fluid is noticed for large values of Ha. The high values of the magnetic parameter (Ha) support the heat transfer irreversibility. Furthermore, throughout the pump it can be observed that the total irreversibility of the system due to fluid friction, ohmic heating and magnetic field overcomes the heat transfer irreversibility. Figure 7b informs that the variations in the Bejan number distribution is insignificant around the pump core area. But when it travels towards the peripheral region, the high values of K assist heat transfer irreversibility. Overall, the heat transfer irreversibility owing to convection and conduction is overshadowed by the system’s total irreversibility. From Fig. 7c,d it is inferred that the high values of the slip parameter (*L*) and radiation number (*R*_n_) aid to the overall system’s irreversibility over the heat transfer irreversibility. Figure 7e clarifies that the Bejan number seems to be an accelerating function of the ohmic heating number (*S*_*p*_). Near the pump core portion, the Bejan number shifts to the origin that communicates to the nil heat transfer irreversibility but in the proximity of the pump surface, the heat transfer results are perceptible.

### Comparative assessment of mono-metallic/bi-metallic/tri-metallic nanofluid

In energy transformation processes, one of the primary challenges is to optimize the efficiency of the thermal system. Such thermal performance is deeply influenced by the thermophysical properties of the working fluids running in considered thermal system. Thus, to investigate of the thermal characteristics of mono nanofluid (TiO_2_/blood), hybrid nanofluid (TiO_2_–SiO_2_/blood), ternary fluid (TiO_2_–SiO_2_–Al_2_O_3_/blood) and traditional base fluid (Carreau fluid model), Fig. 8a–c along with Table 2 are provided. The temperature distributions of all the considered types of nanofluid models is exhibited in Fig. 8a. This figure informs that the ternary fluid preserves enhanced heat transfer characteristics and therefore have elevated temperature compared to hybrid/mono nanofluid and traditional blood. This augmentation arises owing to the interbreeding of different sorts of nanogranules with their distinctive chemical relationships and bonds that add to more heat transfer enrichment. Furthermore, in the contemplated trihybrid fluid, silica (SiO_2_) act as an effective catalyst to assist conduction, Titanium Dioxide (TiO_2_) consists of covalent bonds and therefore endorses convection and Alumina (Al_2_O_3_) keeps chemical inertness, thermal stability and high corrosion resistance.

**Table 2:** The Nusselt number for pure blood, mono nanofluid, bi-hybrid nanofluid, and tri-hybrid fluid at fixed values of Ha = 1, *R*_*n*_ = 2, *U*_*HS*_ =  − 1 and *S*_*p*_ = 0.5

K	Conventional blood	Mono nanofluid	% Increase	Bi-hybrid nanofluid	% Increase	Tri-hybrid fluid	% Increase
1	0.63789	0.67262	5.44	0.69889	9.55	0.70719	10.86
2	0.63096	0.66534	5.44	0.69133	9.56	0.69918	10.81
3	0.62014	0.65358	5.39	0.67883	9.46	0.68565	10.65
4	0.61251	0.64442	5.21	0.66830	9.11	0.67354	9.96
5	0.61165	0.64153	4.86	0.66345	8.47	0.66671	9.00

The available work capability of energy to perform useful work is known as exergy. It means it is the measure of energy transformations to the other forms. During this conversion process, the unusable energy or heat losses are termed as entropy. Figure 8b displays a comparative glance of entropy generation number for hybrid type blood based nanofluids and pure blood. It can be viewed that entropy production inside the electroosmotic pump is minimum if ternary fluid is considered as working fluid. The variations in the Bejan number profile are reported through Fig. 8c. This figure reveals that the ternary fluid has considerable capability to conquer the irreversibility due to heat transfer effects compared to the mono and hybrid nanofluids. To inspect the rate of heat transfer in conventional blood, mono nanofluid, hybrid and ternary nanofluids, Table 2 is provided. It can be noticed that the heat transfer rate is maximum for ternary fluid and minimum for pure blood. For instance, specifically for the values of K = 1, the percentage increase in the heat transfer rate in the ternary fluid is 5.44% more than mono nanofluid, 9.55% higher than bi-hybrid nanofluid and 10.86% up than pure blood.

### Effect of nanoparticles volume fraction

Figure 9a–d is sketched to observe the impact of nanoparticles volume fraction *ϕ* on the temperature profile, heat transfer rate, total entropy number and the Bejan number, respectively. Figure 9a shows that the fluid temperature rises as *ϕ* increases which is obvious result due to increase in thermal conductance of nanofluid. Figure 9b indicates that the heat transfer rate at the surface of pump also increases as *ϕ* augments. Such result is also expected due to the increase in the temperature difference at the surface of pump caused by higher fluid temperature. The total entropy number also increases as the volume fraction *ϕ* increases (see Fig. 9c). The profile of Bejan number shown in Fig. 9d indicates that this increase in the total entropy of the system is mainly caused by the increasing effect of heat transfer irreversibility. Moreover, the Bejan number also shows that heat transfer irreversibility dominates over the other of irreversibilities as *ϕ* rises.

### Streamlines pattern

An appealing feature of fluid stream endorsed by the peristaltic pumping or ciliary motion is termed as trapping. The trapping occurs at elevated amplitude ratio and persistent fluid stream rate. Under certain constraints, a set of closed streamlines divide to restrain a fluid mass. This confined fluid mass named bolus is carried by the streamlines and travels with the same velocity of peristaltic wave or metachronal wave. Whereas as the rest of the fluid travels ahead with less speed than the confined bolus. Figures 10, 11 and 12 reflect this phenomenon for distinct values of the magnetic parameter (Ha), electroosmotic parameters (K) and the Helmholtz–Smoluchowski velocity (*U*_*HS*_). From Fig. 10a,b, it is viewed that the confined mass condenses in size when the elevated values of the magnetic parameter (Ha) are chosen. This diminution in the trapped bolus size is seen due to the impedance of magnetic field to fluid stream rate. Existence of strong EDL (small values of K) effects at the liquid–solid interface decelerate the fluid flow rate and push the imprisoned fluid mass towards the pump edge as portrayed in Fig. 11a,b. Figure 12a,b is plotted to see the impact of the Helmholtz–Smoluchowski velocity (*U*_*HS*_) on streamlines pattern of the ternary fluid. The higher values of *U*_*HS*_ place a significant increment in fluid stream rate of the ternary fluid. This results in expansion of bolus size and generation of new bolus.

## Conclusions

The present work is initiated to investigates the role of ternary nanofluid along with the contribution of momentum slip and inclined magnetic field on thermally radiated electroosmotic pump with flexible walls. The ternary nanofluid is comprised of three distinct types of metallic nano-sized granules of titanium oxide, Silica and Aluminium dioxide blended with pure blood characterized by Carreau fluid model. The internal flexible wall of the channel is lined-up with fabricated cilia. The Carreau nanofluid flow with such settings has never been considered before. The problem is modelled in wave frame of reference and simplified under the lubrication approximation, the Debyeh–Hückel linearization and the Rosseland approximation. The analysis comprises of the comparative investigation of the heat transfer rate and the second-law for simple base-fluid, mono, hybrid and ternary nanofluids. In the light of the above analysis, the following concluding remarks are drawn:

- The ternary fluid showed effective role in enhancing the heat transfer rate as compared to the pure blood, mono/hybrid nanofluid. At a fixed value of electroosmosis parameter, the heat transfer rate is elevated up to 10.86% when compared with the conventional blood model.
- The ternary fluid helps in raising fluid temperature and reduces the heat transfer rate. Thus, it lowers down the total entropy production in the pump.
- The role of thermal radiation is to lessen the fluid temperature which helps in reducing the heat transfer irreversibility and thus the total entropy of the pumps minimizes.
- Momentum slip condition performs an essential part in cooling process by substantially falling the fluid temperature down.
- Around the pump peripheral zone, the elevated values of the Hartmann number and the momentum slip parameter contribute to reinforce the ternary fluid stream.
- The temperature of the ternary nanofluid cools down by considering weak magnetic field and strong EDL effects.
- Heat losses in ternary nanofluid stream can be lessened by integrating strong EDL, thermal radiation and momentum slip effects.

In future such work may be extended to investigate the rheological effects in electroosmotic pump with trimetallic nanofluids through porous or composite channels. Such studies are highly emerging in drug delivery and various medical applications.

## Data Availability

All the data is provided in the manuscript.

## Latin symbols

*a*: Mean width of channel (m)
*B*_0_: Magnetic field (A/m)
*Be*: Bejan number
*c*: Speed of wave transmission (m/s)
*C*_*P*_: Specific heat capacity (J/(kg·K))
*e*: Electric charge (C)
Ec: Eckert number
*E*_*x*_: Applied electric field (V/m)
F: Normalized flow rate (m^3^/s)
Ha: Hartmann number
*k*: Thermal conductivity (W/mK)
*K*: Electroosmotic parameter
*K**: Absorption coefficient (1/m)
*k*_*b*_: Boltzmann constant (J/K)
*L*: Knudsen number
*n*_0_: Average concentration of ions (1/kg)
N_S_: Entropy generation number
*p*: Normalized pressure field (N/m^2^)
$\overline{P}$: Pressure (fixed frame) (N/m^2^)
Pr: Prandtl number
Q: Mean flow rate (m^3^/s)
*R*_*n*_: Radiation parameter
$S_{gen}^{\prime\prime\prime}$: Entropy generation rate (J/K)
S_G0_: Characteristic entropy (J/K)
*S*_*p*_: Joule heating parameter
$$\overline{t}$$: Time variable (s)
$\overline{T}$: Fluid temperature (fixed frame) (K)
T_0_: Reference temperature (K)
T_1_: Wall temperature (K)
*T*_*ave*_: Electrolytic-solution temperature (K)
(*u*, *v*): Dimensionless velocity vector
*U*_*HS*_: Helmholtz–Smoluchowski velocity
($\overline{U},\overline{V}$): Velocity vector V (fixed frame) (m/s)
$\left( {\overline{u}, \overline{v}} \right)$: Velocity vector (moving frame) (m/s)
We: Weissenberg number
X_0_: Particle locale (m)
($\overline{X},\overline{Y}$): Space coordinates (fixed frame) (m)
($\overline{x}, \overline{y}$): Space coordinates moving frame (m)
(*x*, *y*): Dimensionless spatial coordinates
z: Valence of ions

## Greek symbols

α: Eccentricity parameter
*β*: Wave number
ε: Cilia length (m)
$$\epsilon$$: Medium permittivity (C^2^/N-m^2^)
$\epsilon_0$: Permittivity of free space (C^2^/N-m^2^)
*ρ*: Density (kg/m^3^)
Δp_λ_: Pressure rise per wavelength
∆T: Temperature difference (K)
λ: Wavelength (m)
λ_1_: Slip length (m)
*χ*: Directional angle of magnetic field
*θ*: Normalized temperature profile
σ: Electrical conductivity (S/m)
σ*: Stefan-Boltzmann constant
*μ*: Dynamic viscosity (kg/m-s)
*ϕ*: Nanoparticles concentration (mole/m^3^)
$\overline{\Phi }$: Electroosmotic potential function
τ: Normalized temperature difference
*ζ*: Wall electric potential (kg-m^2^/s^3^-A)
Ψ: Stream function

## Subscripts

*thnf*: Trihybrid nanofluid
*hnf*: Binary nanofluid
*e*: Electric charge
*f*: Base fluid
1: Titanium oxide
2: Silica
3: Aluminum oxide
